# WHO Parents Skills Training (PST) programme for children with developmental disorders and delays delivered by Family Volunteers in rural Pakistan: study protocol for effectiveness implementation hybrid cluster randomized controlled trial

**DOI:** 10.1017/gmh.2017.7

**Published:** 2017-06-13

**Authors:** S. U. Hamdani, P. Akhtar, Zill-e-Huma, H. Nazir, F. A. Minhas, S. Sikander, D. Wang, C. Servilli, A. Rahman

**Affiliations:** 1 Human Development Research Foundation, Islamabad, Pakistan; 2 Institute of psychiatry, WHO collaborating centre for mental health research and training Rawalpindi, Pakistan; 3 Liverpool School of Tropical Medicine, Liverpool, UK; 4 Department of Mental Health and Substance Abuse, World Health Organisation, Geneva, Switzerland; 5 University of Liverpool, Liverpool, UK

**Keywords:** Autism spectrum disorders, child mental health, developmental disorders, family volunteers, global mental health, implementation effectiveness hybrid trial, intellectual disability, low income settings, mental health gap, parent skills training, technology assisted training and supervision

## Abstract

**Background.:**

Development disorders and delays are recognised as a public health priority and included in the WHO mental health gap action programme (mhGAP). Parents Skills Training (PST) is recommended as a key intervention for such conditions under the WHO mhGAP intervention guide. However, sustainable and scalable delivery of such evidence based interventions remains a challenge. This study aims to evaluate the effectiveness and scaled-up implementation of locally adapted WHO PST programme delivered by family volunteers in rural Pakistan.

**Methods.:**

The study is a two arm single-blind effectiveness implementation-hybrid cluster randomised controlled trial. WHO PST programme will be delivered by ‘family volunteers’ to the caregivers of children with developmental disorders and delays in community-based settings. The intervention consists of the WHO PST along with the WHO mhGAP intervention for developmental disorders adapted for delivery using the android application on a tablet device. A total of 540 parent-child dyads will be recruited from 30 clusters. The primary outcome is child's functioning, measured by WHO Disability Assessment Schedule – child version (WHODAS-Child) at 6 months post intervention. Secondary outcomes include children's social communication and joint engagement with their caregiver, social emotional well-being, parental health related quality of life, family empowerment and stigmatizing experiences. Mixed method will be used to collect data on implementation outcomes. Trial has been retrospectively registered at ClinicalTrials.gov (NCT02792894).

**Discussion.:**

This study addresses implementation challenges in the real world by incorporating evidence-based intervention strategies with social, technological and business innovations. If proven effective, the study will contribute to scaled-up implementation of evidence-based packages for public mental health in low resource settings.

**Trial registration.:**

Registered with ClinicalTrials.gov as Family Networks (FaNs) for Children with Developmental Disorders and Delays. Identifier: NCT02792894 Registered on 6 July 2016.

## Background

Developmental disorders are lifelong conditions that are characterised by early childhood onset and delay in central nervous system development and maturation. This includes conditions such as autism spectrum disorder and intellectual disabilities (World Health Organization, 1992). Developmental disorders and delays are a public health priority and are included in the World Health Organisation mental health Gap programme (WHO mhGAP) to bridge the treatment gap for priority mental health conditions in low resource settings (Dua *et al*. 2011). WHO mhGAP guidelines recommend Parents Skills Training (PST) for developmental disorders and delays.

Based upon the findings of a systematic review and meta-analysis of literature WHO has developed a PST programme for children with developmental disorders and delays (Reichow *et al*. 2013). Two key features of WHO PST are (a) the programme takes a task-shifting approach where non-specialists including social workers, nurses, teachers, community volunteers, caregivers and parents can deliver this programme in community-based facilities or schools to the families of children with developmental disorders and delays (b) it takes a trans-diagnostic approach – it addresses a range of different developmental disorders and conditions and does not require an expert diagnosis of developmental disorders and delays to qualify treatment. However fragile health systems, lack of funding and lack of trained health personnel remain the key bottlenecks to scale up and sustainability of such priority public health intervention packages in low resource settings (Eaton *et al*. 2011).

**Fig. 1.**
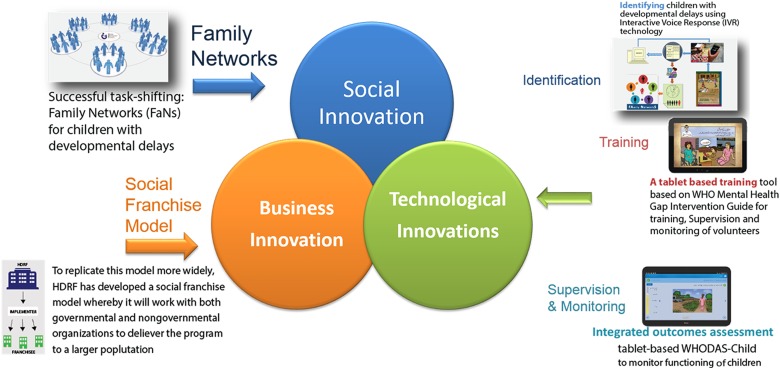
Integrated model of service delivery.

To overcome these challenges, we developed an integrated model of service delivery (incorporating social, technological and business innovations (Fig. 1) for children with developmental disorders and delays in low resource settings (Hamdani *et al*. 2015). An android application was developed to incorporate the WHO mhGAP-IG diagnostic and management guidelines and WHO PST for developmental disorders and delays in a standardised way. The intuitive tablet-based training and intervention delivery tool can be used to deliver evidence-based interventions in low resource settings by non-specialist. The model was found to be feasible, acceptable and resulted in change in client outcomes. The proof of concept of the integrated model of service delivery has already been published (Hamdani *et al*. 2015).

### Objectives and hypothesis

The objectives of this study are to evaluate at scale the effectiveness and implementation of WHO PST programme for children with developmental disorders and delays in rural Pakistan.

To obtain the objectives of the study, following research questions are formulated; (1) Can the WHO PST delivered by family volunteers improve functioning in children with development disorders and delays in rural Pakistan? (2) Can the WHO PST programme improve child's social communication and joint engagement with caregivers, improve child's social and emotional well-being, improve family empowerment, and result in better caregiver's health related quality of life? (3) Can the integrated model of service delivery for developmental disorders and delays (Hamdani *et al*. 2015) serve as a scalable method of delivering WHO PST programme in low resource settings? (4) What factors can inhibit and promote the large-scale implementation of WHO PST programme for developmental disorders and delays?

The primary effectiveness hypothesis is that WHO PST programme for children with developmental disorders and delays plus Enhanced Treatment as Usual (ETAU) is superior to ETAU alone in improving functioning in children with developmental disorders and delays in rural Rawalpindi.

Secondary effectiveness hypotheses are that WHO PST programme for children with developmental disorders and delays is associated with improvements in child's social communication and joint engagement with the caregiver, Child's social and emotional well-being, family empowerment, care giver's health related quality of life and reduction in family's stigmatizing experiences.

Primary implementation hypothesis is that the integrated model of service delivery for WHO PST program is a feasible, acceptable, appropriate implementation strategy that results in fidelity, adoption and penetration of the parents’ skills training program and increases the reach of evidence-based services for families and children with development disorders and delays in low resource settings.

## Methods

### Study settings

Study will be conducted in 30 rural Union Councils (UCs)-the smallest administrative units- of sub-district Gujar Khan in Rawalpindi district, Pakistan (approximate population 1 million). Sub-district Gujar Khan, representative of northern Punjab and Pothohar region, is a rural sub-district situated about 35 km south east of the Rawalpindi city.

Each UC has a Basic Health Unit (BHU) that provides health care to local population. BHU is staffed by a medical doctor, a dispenser, 15–20 Lady Health Workers (LHWs) working under supervision of Lady Health Supervisor, Lady Health Visitor, and a Vaccinator. Nearest specialist mental health care facility is the Institute of Psychiatry, Rawalpindi. One public special education school for children with special needs caters to the needs of children with all kinds of special needs in Gujar Khan.

### Design

The trial is defined as an effectiveness implementation hybrid cluster randomised trial-type II because the focus of evaluation is the effectiveness of intervention delivery and implementation strategies at scale. This is the appropriate design to use because there is evidence to support the feasibility, fidelity, acceptability, appropriateness of the implementation strategies in the proposed settings (Hamdani *et al*. 2014, 2015). There is evidence on the effectiveness of WHO PST program strategies (Reichow *et al*. 2013; Rahman *et al*. 2016*a*). However, there is no evidence on the effectiveness of intervention and implementation strategies at scale. This will be the first study to evaluate the effectiveness of scaled up implementation of WHO PST program in real world settings.

The first and second research questions will be answered by single blind cluster randomised control trial (cRCT) with two arms. Implementation effectiveness will be evaluated using the updated implementation research framework of Proctors *et al*. (2011) and the RE-AIM framework (http://re-aim.org/). We will use the Applied Mental Health Research Dissemination and Implementation (AMHR D&I) measurements to collect data at organization, provider and consumer levels post program implementation (AMHRG, personal communication). We will calculate the RE-AIM basic and summary indices on implementation outcomes. Quantitative and qualitative techniques will be utilised to supplement the implementation data. Data will be gathered from the implementation checklists (number of family volunteer trained, number of Family Networks established, tracking attendance of sessions and implementation and intervention fidelity) completed during the trial and through process evaluation during and after intervention delivery.

A cRCT design having UC as the unit of randomisation was preferred. Randomising smaller units, for example villages within the UCs, would lead to contamination and spillage of information in between the intervention and control villages. Hence, randomising a UC could reduce the probability of contamination between the two groups.

## Interventions

### WHO PST programme delivered by family volunteers

The core of the intervention is the WHO PST programme delivered within the broader context of WHO mhGAP programme implementation in Pakistan. WHO PST programme for children with developmental disorders and delays (field trial version 1.0) consists of eight sessions delivered in group format by a lay-facilitator. The PST Programme aims to provide evidence-based PST for caregivers of children with developmental disorders and delays that can be implemented locally in low- and middle-income countries. The primary aims of the programme are to promote better understanding and acceptance of developmental disorders and developmental delays and help parents apply skills that promote child development, communication and functioning. The secondary aims of the programme include strengthening caregivers’ coping skills and psychological well-being, and promoting child adaptive behaviours. It is expected that the programme will facilitate stigma reduction against persons with developmental disorders and result in increased inclusion and participation of children with developmental disorders and delays. The WHO PST programme is available on request.

**Fig. 2.**
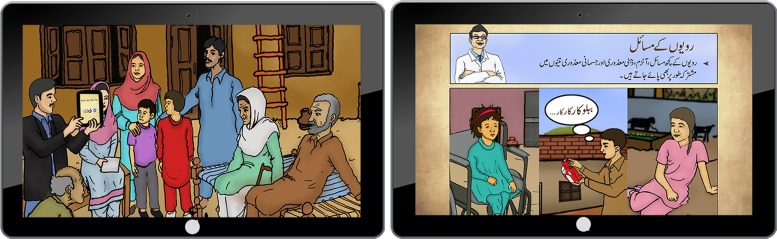
Avatar-Assisted Cascade Training (ACT).

Following the WHO PST adaptation guide, we adapted the pre-publication draft of the WHO PST programme for children with developmental disorders and delays for implementation in rural Rawalpindi, Pakistan. The adapted WHO PST programme for children with developmental disorder and delays delivered by family volunteers *consists* of nine weekly sessions. It is delivered by a ‘champion’ family volunteer in group settings through the use of intuitive tablet-based android application that serves as a training, intervention-delivery and monitoring tool. The key psycho-educational contents (‘key messages’ and strategies) of PST have been incorporated into ‘real-life’ narratives of the lives of three children with developmental disorders, their family members, and other supporting characters. An artist has converted the characters into ‘Avatars’ (graphic image representing each character) (Fig. 2), which are used to voice the narrative scripts. The details of the service delivery model and training, supervision and monitoring tool have been described elsewhere (Hamdani *et al*. 2015).

Providers of PST programme for children with developmental disorders and delays are ‘champion’ family volunteers. These are parents or caregivers of children with developmental disorders and delays, have at least eight grades of formal education, are voluntarily willing to be trained and supervised by the trainers for at-least 6 months duration of the programme and cascade the training to 4–5 families in their villages. Our earlier work has established that lay health workers can deliver evidence-based psychological interventions in Pakistan (Rahman *et al*. 2008, 2016*b*). The training and supervision of champion family volunteers will follow a cascade model (Murray *et al*. 2011). The training in the WHO PST will be delivered using an android application hosted on a tablet device. The master trainers will train 10 trainers in the WHO PST programme. The training will consist of 10 days class-room training followed by case studies under the supervision of master trainer. The master trainer will perform live competency rating of the trainers on the case studies using an adapted version of ENhancing Assessment of Common Therapeutic factors (ENACT) for PST for developmental disorders and delays (Kohrt *et al*. 2015). The trainers have all completed at-least Masters in Clinical Psychology and have at-least 1 year of experience in working with children and families with developmental disorders and delays.

Only competent trainers (having mean score of 2.5 on each domain of ENACT adapted for WHO PST programme competency and fidelity rating) will be allowed to train and supervise champion family volunteers. The trainers will cascade down the training to the champion family volunteers using tablet-based tool, who will, then, deliver the PST programme to the 4–5 families under their care in local villages.

The champion family volunteers will be supervised by the trainers, who will be supervised by the master trainers on monthly basis. The fidelity of program delivery will be established by the master trainers by rating a random sample of sessions using the ENACT adapted for WHO PST program.

### Enhanced treatment as usual

Treatment as usual in primary healthcare centres in Rawalpindi for childhood developmental disorders usually consists of no treatment, or a range of alternate treatment regimes, such as multi-vitamin syrups and tablets. Evidence-based mental health care is currently not available in primary healthcare centres. For this study, the treatment as usual is enhanced in two ways:(a) LHWs in the ETAU arm will receive training in recognising signs and symptoms of developmental disorders and delays and making referrals to their primary care physicians for treatment; and (b) These primary care physicians will receive training in mhGAP developmental disorders standard module by the specialist at Institute of Psychiatry, WHO Collaborating Center for mental health in Pakistan.

If, during this treatment or during the study's assessments participants in ETAU arm show severe psychiatric disorders (e.g. psychosis, uncontrolled epilepsy) or problems (e.g. behavioural problem) that require immediate specialist treatment and follow-up, they will be referred to the child mental health facility at the Institute of Psychiatry, WHO Collaborating Center. The ETAU group will receive treatment when both arms will complete their follow up evaluation.

### Participants’ recruitment

#### Inclusion criteria

Children aged between 2 and 12 years residing in the study area for the duration of the study will be invited to participate in the trial. Inclusion criteria are as follows:

- Children who are screen positive on any of the Ten Questions Screen questionnaire items # 1,4,5,7,8,9,10 for neurodevelopmental delay (Durkin *et al*. 1995)
- Diagnosed as developmental disorder/delays according to clinical assessment (History & clinical examination for developmental delay in motor, communication, social, cognitive, daily living skills domains according to mhGAP developmental disorders guidelines for clinical assessment in primary healthcare settings.

#### Exclusion criteria

The exclusion criteria are;

- Co-morbid physical or mental conditions in the child requiring inpatient hospitalisation
- Deafness or blindness in the child or caregiver.
- Primary caregiver not available or unwilling to participate in the intervention programme for 6 months
- Physical or mental conditions in the primary caregiver needing inpatient hospitalisation or frequent outpatient visits (more than two times a month).
  All these conditions will be observed by the trained assessor and will be inquired from the care-givers and family members using a checklist.

### Procedure to identify eligible participants

Prior to randomisation, key stakeholders (village elders and the primary health care staff) will be informed about the study procedures and their permission will be sought. HDRF has a database of about 3000 families and children with developmental disorders and delays gathered by interactive voice response (Hamdani *et al*. 2015) and snowballing sample technique as a part of the Foundation's service delivery to the community.

For the purpose of the trial we will need to identify 540 parent-child dyads from within 30 clusters (18 parent-child dyads from each cluster) to complete baseline and endpoint assessments. However according to the existing database of children, we expect that in each UC/cluster there will be more than 18 children fulfilling the eligibility criteria. For the purpose of the trial, within each UC, evaluation zones will be made to assess the impact of PST programme for children with developmental disorders and delays delivered by family volunteers.

These evaluation zones will be made by randomly selecting one LHW out of the total 15–20 LHWs serving a particular UC. The random selection will be carried out by an independent researcher not involved in the trial using a simple random table. If the required number of dyads is not completed from one LHW catchment area, we will include the catchment areas of adjacent LHW to reach the required sample size from each UC. This patch of contiguous area/catchment areas of LHWs within that UC will serve as the impact evaluation zone for our trial related outcomes.

### Randomisation

As it is a cRCT, unit of randomisation is cluster. Each cluster is the smallest administrative unit, called UC. Thirty UC clusters will be randomised to the intervention and control arms on a 1:1 allocation ratio using a permuted-block randomisation method. SAS PROC PLAN is carried out to generate a randomisation list by an independent statistician based at University of Liverpool. Allocation of clusters will be carried out by an independent person not involved in the conduct of the trial.

### Informed consent

The trained research team along with LHW will approach the families within the evaluation zones of the UCs to seek informed consent for participation in the trial. For the families who consent for participation in the trial, the research team will evaluate caregiver-child dyad against trial inclusion and exclusion criteria. Baseline assessments will be completed for consenting parent-child dyads. LHW will inform the families about the allocation status. Families randomised to intervention arm will be organised into networks with one champion family volunteer for 5–7 families. A record of outcome of screening, assessment against eligibility criteria, and reasons for refusal will be maintained for those who do not consent for participation in the trial. At the endpoint, researcher will verbally reaffirm the consent.

### Sample and power calculations

The effect size estimates for the primary outcome measure (WHODAS-Child) are informed by the results of the pre-post feasibility study in Pakistan (Hamdani *et al*. 2015) and are in line with the effect sizes reported in a recent WHO supported systematic review of literature for non-specialist delivered psychosocial interventions for developmental disorders in lower middle income countries (Reichow *et al*. 2013).

We aim for a conservative effect size estimate of 0.35 for the primary and secondary outcome measures in the definitive cluster randomised trial.

We propose to recruit a sample of 540 parent-child dyads in 30 clusters (on average 18 parent-child dyad per cluster), equally distributed among the intervention and control arms. This will give 93% power at 5% two sided significance level with an ICC of 0.01 and will account for 15% attrition rate.

Since the study addresses developmental disorders and delays, which are manifested in multiple ways, we expect the clusters to be heterogeneous with respect to diagnosis, severity, age and co-morbid conditions, hence we have chosen a lower value of ICC of 0.01.

### Outcomes evaluation

Baseline and end point assessments (6 months post intervention) on the sample of 540 parent-child dyads will be conducted by the trained research assessment team blind to the allocation status of the trial participants. Research assessment team is trained in administration of all outcome assessment tools, interviewing techniques, and principles of good clinical practices. The assessments will be completed at a venue of participant's choice-either at their own house, or the LHWs house called the ‘Health House’ or at any other household within the community.

### Primary intervention effectiveness outcome

#### World Health Organization Disability Assessment Schedule Child Version (WHODAS- Child 2.0)

The WHODAS is a 36-item questionnaire measuring functioning and disability. The items represent cognition, mobility, self-care, getting along with others, life activities, and participation in the society. The interviewer administered proxy version (caregiver report) will be used as it is expected children in this population would not be able to self-report. The tool has been translated, culturally adapted and validated for children with developmental disorders in Pakistan and indicted satisfactory psychometric properties in terms of internal consistency, construct validity and factor structure (*Hamdani et al. forthcoming*).

### Secondary intervention effectiveness outcomes

1. *Caregiver–Child Interaction* (Baggett *et al*. 2009): Fifteen minute video taped caregiver–child interactions will be collected at baseline and at endpoint for families in both arms of the study. Caregivers will be asked to try home routines involving the child (e.g. feeding the child performing domestic chores) or play based routines (e.g. playing with toys or reading a book) with their child. Caregiver's facilitators and interrupters and child's engagement and distress during social communication and joint engagement will be rated. The CCI videos will be singly coded by trained assessors.
2. *Clinical Global Impressions* (**McConachie *et al*. 2015**): CGI ratings for focusing on the child's social communication challenges will be conducted at baseline and endpoint. The CGI ratings include two scores: (a) severity of challenges in social communication (rated from 1–7) and (b) improvement in social communication from entry to exit (rated from 1–7).
3. *Socio emotional well-being of children* (Goodman, **1997**): Strength and Difficulties Questionnaire (SDQ) is a Parent rated, 25 items scale distributed over five domains: emotional symptoms; conduct problems; hyperactivity/inattention; peer relationship problems, and; prosocial behavior. Each item is rated on a 3-point Likert scale (0 = not true, 1 = somewhat true, 2 = certainly true). Total difficulty score is calculated by adding the scores of all domain except prosocial behavior items (Goodman, 1997). SDQ has been validated in Pakistani and has shown good psychometric properties (Samad *et al*. 2005; Syed *et al*. 2007).
4. *Parental health related quality of life* (**Varni *et al*. 1999**): Parental health related quality of life will be measured by Pediatric Quality of Life (Peds-QL) Family impact module. The Peds-QL is 36 item impact module scale that encompasses 6 sub-scales measuring parent self-reported functioning. These subscales measure physical functioning, emotional functioning, social functioning, daily activities and family relationships. Items are rated on a 5 point Likert scale (0 = never to 4 = almost always). Peds-Ql has shown sound psychometric properties in different cultures (Scarpelli *et al*. 2008; Chen *et al*. 2011).
5. *Family empowerment measured by Family Empowerment Scale (FES)* (**Koren *et al*. 1992**): The family empowerment scale (Koren *et al*. 1992) is a parent rated, 34 items scale consisting of three subscales. The family subscale (12 items) refers to the parents’ management of everyday situations. The service system subscale (12 items) refers to parents’ acting to obtain services to meet the child's needs. The community subscale (10 items) refers to parents’ advocacy for improving services for children in general. Each item is rated on a 5-point Likert scale (1 = not true at all to 5 = very true). Scores are summed across all items for each subscale with higher scores indicating relatively more empowerment. FES has shown good internal consistency, test-retest reliability and factor structure (Koren *et al*. 1992).
6. *Caregivers’ stigmatizing experiences measured by Inventory of Stigmatising Experiences (ISE)* (Stuart *et al*. 2005): This is an interview based measure of the extent of stigma faced by family. Consisting of Seven items, each item is rated on a 5-point liker scale (1 = never to 5 = always). The responses are recoded into a binary variable with 1 reflecting presence of stigma and 0 reflecting absence of stigma. Scores are summed across all items with a maximum score of 7, with lower scores indicating relatively less stigma. The scale indicated good internal consistency (Stuart *et al*. 2005).
7. *Life Events and Social Factors*: A translated and culturally adapted version of Life Events and Disability Schedule (LEDS) (Husain *et al*. 2000) will be administered at follow up to measure the exposure to any potential negative life event/s that happened since the baseline assessment.

### Implementation effectiveness outcomes

1. *Applied Mental Health Research Dissemination and Implementation measurements (AMHRG, Personal communication)*: The effectiveness of implementation will be measured by an adapted version of AMHR D& I measurement tool. The consumer, provider and organizational versions of instrument will be administered at 6 months follow up. The consumer instrument consists of scales to measure Acceptability (17 items), Adoption (12 items), Appropriateness (13 items), Feasibility (14 items), and Penetration (8 items).

The provider level instrument contains 16 items to measure Acceptability, 9 items for Adoption, 16 items for Appropriateness, 20 items for Feasibility, 8 items for Penetration and additional scales to measure Organisational Climate (13 items), and Organisational Leadership (10 items).

At the organisational staff level there are 10 Acceptability items, 13 Adoption items, 12 Appropriateness items, 14 Feasibility items, 8 Penetration items, 15 Organisational Climate items and 10 Organisational Leadership items. Each item is scored on a 4-point ordinal scale ranging from 0 ‘not at all’ to 3 ‘a lot,’ with an additional category for ‘don't know/not applicable.’

The tool was tested using mixed methods as part of a project focused on scale-up of evidence-based mental health programs in Iraqi Kurdistan and Myanmar. Reliability results showed adequate internal consistency reliabilities (ranged from 0.61 to 0.95).

1. *Cost*: The cost of implementing the WHO PST Program delivered by Family volunteers at scale will be calculated using the project financial data. Service utilization and out of pocket expenditure of the study participants (costs for: seeing a doctor or other health care providers; admission to hospital, medicines, tests and extra help at home needed, the utilization of various health and social care services including time and opportunity losses by the families in the care of their child with developmental delay/disorder) will be collected in the trial at baseline and 6 months follow up. Health services utilisation by the trial participants will be measured using the Client Service Receipt Inventory (Chisholm *et al*. 2000) that has been adapted for use in children and families with developmental disorders and delays.

### Masking

Given the nature of WHO PST programme intervention, it is not possible to mask the participants and facilitators, as well as the qualitative research team staffs. All researchers conducting pre-post quantitative outcome assessments will be masked in the trial. The order of outcome measures ensures that the primary outcome measure (WHODAS) is administered first as this will minimise the risk of bias that can happen if masking is compromised. If unmasking does occur this will be documented, the assessment halted, and a new researcher assigned to complete assessments with that participant.

The trial statistician will also be blinded regarding the treatment code when he develops the statistical analysis plan and writes the statistical programmes, which will be validated and completed using dummy randomisation codes. The actual allocation will only be provided to the study team after lock of the database.

### Data management

*Quantitative data*: The pre-post data from study participants will be collected by the assessment team using hand held devices (Tablets) with pre-assigned participant codes. Database will be stored in password protected computers and backed-up on daily basis. The data will be downloaded into SAS and SPSS formats for statistical analysis.

*Qualitative data*: Anonymised data will be stored in paper format in locked filing cabinets in the field office.

### Statistical methods

**Fig. 3.**
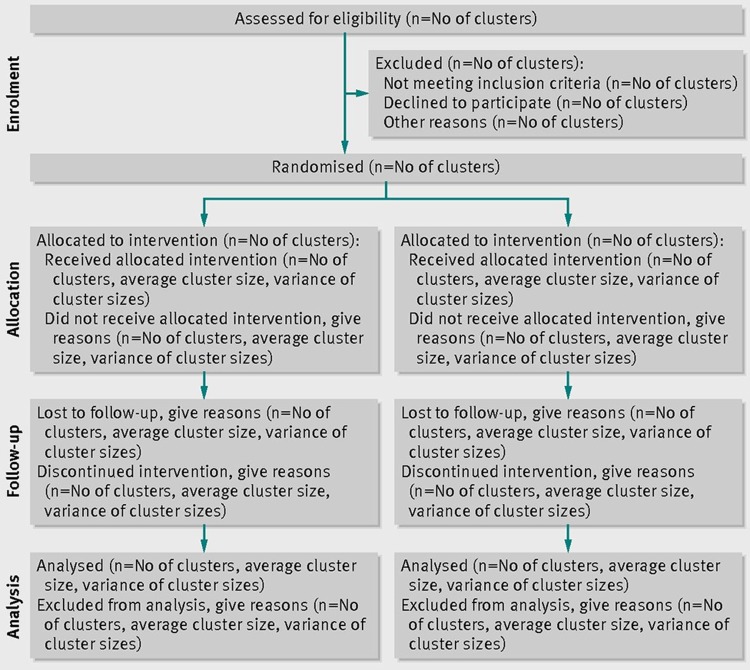
CONSORT flow chart.

Findings of the trial will be reported following the recommendations of the CONSORT 2010 statement: extension to cluster randomised trials (Schulz *et al*. 2010) (See Fig. 3).

All the analysis will be based on intention to treat. Data will be analysed using SAS 9.3 and SPSS Version 21. To estimate the treatment effect, a linear mixed model will be employed for the primary endpoint analysis, which will have treatment as fixed effect, and baseline measurement of primary endpoint as covariate, and cluster as random effect. The mean difference between two treatment arms at 6 months together with its 95% confidence interval will be derived from the mixed model. Covariate-adjusted mixed model of primary endpoint will also be performed by adding pre-specified covariates at baseline into the above model. Subgroup analysis will also be performed for the pre-specified covariates used in the covariate adjusted analysis.

### Qualitative and quantitative methods to answer research questions 3 and 4

The updated implementation research framework by Proctor *et al*. (2011) have identified eight key constructs of implementation research. We will collect data on the implementation outcomes at 6 months post program implementation using an adapted version of AMHR D&I measurement tool. Other implementation indicators that will be collected during the program delivery include

- Number and duration of sessions delivered: Information will be collated from the log books of the conducted sessions, which will record basic information about the session – the date, time, and duration of the session.
- Number of participants who completed the treatment and have a planned discharge.
  Number of Family Networks established by family volunteers within their allocated vicinity.

### Process evaluation

Qualitative process evaluation will be conducted to explore the factors promoting and inhibiting the large-scale implementation of the model such as what are potential barriers and facilitators to real-world implementation, sustainability and scalability of the intervention? What problems were associated with delivering the intervention during the clinical effectiveness trial and how will they translate or not to real-world implementation? What modification could be made to the clinical intervention to maximise implementation and sustainability? What potential implementation strategies appear useful or not in ensuring a sustainable and scalable method for delivery PST in low resource setting? These factors will be explored by in-depth interviews and focus group discussion with a sub-sample of study participants including completers, non-completers and drop outs, intervention trainers, supervisors, Health care staff, and family volunteers. Focus groups and interviews will follow an interview guide to cover all the above areas. Sampling will be determined by saturation. The qualitative data will be analysed using the framework analysis approach.

### Ethical considerations

Trial received ethical approval from institutional review board Human Development Research Foundation, Islamabad, Pakistan. As the study deals with the children with developmental disorders and delays, informed consent will be sought from parents. Confidentiality/anonymity of the participants will be protected.

### Dissemination of results and publication policy

Results of the study will be disseminated in English scientific peer-reviewed journals as well as in Urdu (national language) to key stakeholders (e.g. heads of relevant health services, participants in the community engagement meetings, provisional and national governments). In case this cRCT establishes the effectiveness of WHO PST delivered by family volunteers, it will be made widely available as part of WHO's flagship mhGAP programme.

### Trial management

Trial management will be provided by Project Steering Committee that comprises senior research and intervention staff, the PI, technical experts and external advisors who meet Quarterly. The Project Steering Committee receives and reviews reports from the Trial Management Committee (TMC) that comprises the PI, local research and intervention team leads responsible for day-to-day conduct of the trial. The TMC meets on a weekly basis to review the progress of the trial against the trial protocol.

### Data monitoring committee

This committee comprises the PI, assessment team lead and data management team responsible to check data for consistency and completeness.

### Adverse events (AE) reporting

All the AE and serious AE will be recorded by research or intervention staff and will be reported to an independent local trial advisory board. The trial advisory board will review all AE twice a month and will determine if any appropriate action in respect of ongoing trial conduct is necessary and specify what action this would be (i.e. referral to specialised care, suspension of intervention etc.).

## Discussion

WHO PST for developmental disorders and delays is being made available publically through the highest policy platform of the World Health Organisation. However, robust scientific evaluation of the programme and its implementation strategy is mandatory before making it publically available. The WHO PST is being tested in 13 different sites across the globe including Pakistan. This is the first definitive evaluation of the WHO PST Programme in low resource setting. The current study is unique in that it incorporates evidence-based intervention strategies with social, technological and business innovations for sustainable and scalable delivery in real world settings. The study takes the form of a robust implementation evaluation hybrid cRCT design. If proven effective the model will contribute to the knowledge of at-scale and sustainable delivery of public mental health intervention programmes in low resource settings.

### Trial status

Trial recruitment commenced in March 2016 and at the time of manuscript submission the trial was ongoing. Results of this study are expected in December 2017.

## References

[ref1] Baggett KM, Carta JJ, Horn EM (2009). Indicator of Parent-Child Interaction (IPCI) User's Manual. Department of Special Education, University of Kansas, University of Kansas, USA.

[ref2] Chen R, Hao Y, Feng L, Zhang Y, Huang Z (2011). The Chinese version of the Pediatric Quality of Life Inventory™(PedsQL™) family impact module: cross-cultural adaptation and psychometric evaluation. Health and Quality of Life Outcomes 9, 16. 21429195 10.1186/1477-7525-9-16 PMC3072920

[ref3] Chisholm D, Knapp MR, Knudsen HC, Amaddeo F, Gaite L, Van Wijngaarden B (2000). Client socio-demographic and service receipt inventory-European version: development of an instrument for international research EPSILON Study 5. British Journal of Psychiatry 177, s28–s33. 10.1192/bjp.177.39.s28 10945075

[ref4] Dua T, Barbui C, Clark N, Fleischmann A, Poznyak V, Van Ommeren M, Yasamy MT, Ayuso-Mateos JL, Birbeck GL, Drummond C (2011). Evidence-based guidelines for mental, neurological, and substance use disorders in low-and middle-income countries: summary of WHO recommendations. PLoS Medicine 8, e1001122. 22110406 10.1371/journal.pmed.1001122 PMC3217030

[ref5] Durkin MS, Wang W, Shrout PE, Zaman SS, Hasan Z, Desai P, Davidson L (1995). Evaluating a ten questions screen for childhood disability: reliability and internal structure in different cultures. Journal of Clinical Epidemiology 48, 657–666. 7537327 10.1016/0895-4356(94)00163-k

[ref6] Eaton J, Mccay L, Semrau M, Chatterjee S, Baingana F, Araya R, Ntulo C, Thornicroft G, Saxena S (2011). Scale up of services for mental health in low-income and middle-income countries. Lancet 378, 1592–1603. 22008429 10.1016/S0140-6736(11)60891-X

[ref7] Goodman R (1997). The strengths and difficulties questionnaire: a research note. Journal of Child Psychology and Psychiatry 38, 581–586. 9255702 10.1111/j.1469-7610.1997.tb01545.x

[ref8] Hamdani SU, Atif N, Tariq M, Minhas FA, Iqbal Z, Rahman A (2014). Family networks to improve outcomes in children with intellectual and developmental disorders: a qualitative study. International Journal of Mental Health Systems 8, 7. 24485093 10.1186/1752-4458-8-7 PMC3915557

[ref9] Hamdani SU, Minhas FA, Iqbal Z, Rahman A (2015). Model for service delivery for developmental disorders in low-income countries. Pediatrics 136, 1166–1172. 26598452 10.1542/peds.2015-0861

[ref10] Husain N, Creed F, Tomenson B (2000). Depression and social stress in Pakistan. Psychological Medicine 30, 395–402. 10824659 10.1017/s0033291700001707

[ref11] Kohrt BA, Jordans MJ, Rai S, Shrestha P, Luitel NP, Ramaiya MK, Singla DR, Patel V (2015). Therapist competence in global mental health: development of the ENhancing Assessment of Common Therapeutic factors (ENACT) rating scale. Behaviour Research and Therapy 69, 11–21. 25847276 10.1016/j.brat.2015.03.009 PMC4686771

[ref12] Koren Pe, Dechillo N, Friesen BJ (1992). Measuring empowerment in families whose children have emotional disabilities: a brief questionnaire. Rehabilitation Psychology 37, 305–321.

[ref13] Mcconachie H, Parr JR, Glod M, Hanratty J, Livingstone N, Oono IP, Robalino S, Baird G, Beresford B, Charman T (2015). Systematic review of tools to measure outcomes for young children with autism spectrum disorder. Health Technology Assessment 19(41). 10.3310/hta19410 PMC4781156 26065374

[ref14] Murray LK, Dorsey S, Bolton P, Jordans MJ, Rahman A, Bass J, Verdeli H (2011). Building capacity in mental health interventions in low resource countries: an apprenticeship model for training local providers. International Journal of Mental Health Systems 5, 1. 22099582 10.1186/1752-4458-5-30 PMC3284435

[ref15] Proctor E, Silmere H, Raghavan R, Hovmand P, Aarons G, Bunger A, Griffey R, Hensley M (2011). Outcomes for implementation research: conceptual distinctions, measurement challenges, and research agenda. Administration and Policy in Mental Health and Mental Health Services Research 38, 65–76. 20957426 10.1007/s10488-010-0319-7 PMC3068522

[ref16] Rahman A, Divan G, Hamdani SU, Vajaratkar V, Taylor C, Leadbitter K, Aldred C, Minhas A, Cardozo P, Emsley R (2016 *a*). Effectiveness of the parent-mediated intervention for children with autism spectrum disorder in south Asia in India and Pakistan (PASS): a randomised controlled trial. Lancet Psychiatry 3, 128–136. 26704571 10.1016/S2215-0366(15)00388-0

[ref17] Rahman A, Hamdani SU, Awan NR, Bryant RA, Dawson KS, Khan MF, Azeemi MM-U-H, Akhtar P, Nazir H, Chiumento A (2016 *b*). Effect of a multicomponent behavioral intervention in adults impaired by psychological distress in a conflict-affected area of Pakistan: a randomized clinical trial. JAMA Journal of the American Medical Association 316 (24). 10.1001/jama.2016.17165 27837602

[ref18] Rahman A, Malik A, Sikander S, Roberts C, Creed F (2008). Cognitive behaviour therapy-based intervention by community health workers for mothers with depression and their infants in rural Pakistan: a cluster-randomised controlled trial. Lancet 372, 902–909. 18790313 10.1016/S0140-6736(08)61400-2 PMC2603063

[ref19] Reichow B, Servili C, Yasamy MT, Barbui C, Saxena S (2013). Non-specialist psychosocial interventions for children and adolescents with intellectual disability or lower-functioning autism spectrum disorders: a systematic review. PLoS Medicine 10, e1001572. 24358029 10.1371/journal.pmed.1001572 PMC3866092

[ref20] Samad L, Hollis C, Prince M, Goodman R (2005). Child and adolescent psychopathology in a developing country: testing the validity of the strengths and difficulties questionnaire (Urdu version). International Journal of Methods in Psychiatric Research 14, 158–166. 16389892 10.1002/mpr.3 PMC6878532

[ref21] Scarpelli AC, Paiva SM, Pordeus IA, Varni JW, Viegas CM, Allison PJ (2008). The Pediatric Quality of Life Inventory™(PedsQL™) family impact module: reliability and validity of the Brazilian version. Health and Quality of Life Outcomes 6, 35. 18492262 10.1186/1477-7525-6-35 PMC2412859

[ref22] Schulz KF, Altman DG, Moher D (2010). CONSORT 2010 statement: updated guidelines for reporting parallel group randomised trials. BMC Medicine 8, 1. 21350618 10.4103/0976-500X.72352 PMC3043330

[ref23] Stuart H, Milev R, Koller M (2005). The inventory of stigmatizing experiences: its development and reliability. World Psychiatry 4, 35–39.

[ref24] Syed EU, Hussein SA, Mahmud S (2007). Screening for emotional and behavioural problems amongst 5–11-year-old school children in Karachi, Pakistan. Social Psychiatry and Psychiatric Epidemiology 42, 421–427. 17450455 10.1007/s00127-007-0188-x

[ref25] Varni JW, Seid M, Rode CA (1999). The PedsQL™: measurement model for the pediatric quality of life inventory. Medical Care 37, 126–139. 10024117 10.1097/00005650-199902000-00003

[ref26] World Health Organization (1992). The ICD-10 Classification of Mental and Behavioural Disorders: Clinical Descriptions and Diagnostic Guidelines. World Health Organization: Geneva.

